# Editorial: Psychology and art: exploring new ways of interaction

**DOI:** 10.3389/fpsyg.2025.1542235

**Published:** 2025-01-27

**Authors:** Flavia De Simone, Panagiotis Kourtesis, Simona Collina, Francesca Nicolais, Roberta Presta, Roberto Montanari

**Affiliations:** ^1^Scienza Nuova Research Centre, Suor Orsola Benincasa University, Naples, Italy; ^2^Department of Psychology, The American College of Greece, Athens, Greece; ^3^Department of Psychology, National and Kapodistrian University of Athens, Athens, Greece; ^4^Department of Psychology, The University of Edinburgh, Edinburgh, United Kingdom

**Keywords:** psychology, art, interdisciplinary, emotion, artificial intelligence, generative art, aesthetic experience, therapy

The relationship between art and psychology has long been a subject of investigation, standing at the crossroads of creative expression and scientific exploration of the human mind.

This connection not only has historical roots but continues to evolve through modern disciplines such as neuroaesthetics and art therapy, which provide tools to understand and address the complexities of the human condition. Freud himself acknowledged the potential of art to externalize the unconscious, foreshadowing the central role of artistic expression in emotional regulation and internal dialogue. Today, the intersection between art and psychology transcends historical narratives to encompass a neuroscientific investigation of the brain's responses to art and its therapeutic implications.

Neuroaesthetics explores the neural mechanisms underlying artistic perception and creation. Recent studies demonstrate that the aesthetic experience engages a wide range of cognitive and emotional processes, activating specific brain regions such as the superior temporal sulcus and the posterior cingulate cortex, which are respectively responsible for perceptual analysis and emotional reflection (Baluku, 2024).

In parallel, art therapy leverages creative processes to stimulate neuroplasticity, promoting structural and functional changes in the brain that enhance emotional wellbeing and cognitive resilience (Strang, 2024). Together, these disciplines underscore the dual significance of art as both a subjective experience and a scientifically grounded therapeutic tool.

One of the most compelling aspects of this intersection is how art bridges the conscious and unconscious realms of the mind. Creative engagement has been shown to increase synaptic density and activate neural circuits associated with emotional processing, thereby fostering emotional resilience (Strang, 2024). At the same time, neuroaesthetic research elucidates how the brain processes artistic stimuli by integrating perceptual, cognitive, and emotional networks. This integration not only enhances our understanding of the individual's aesthetic experience but also highlights art's profound psychological impact (Baluku, 2024).

These findings transcend theoretical interest, offering tangible applications in therapeutic practices and environmental design. By incorporating aesthetic principles into psychological interventions, practitioners can create tools and spaces that promote both emotional health and cognitive functionality. Understanding the neural underpinnings of artistic experiences is essential for advancing evidence-based approaches that harness the therapeutic and transformative power of art.

The relationship between art and psychology is not only an interdisciplinary curiosity but a robust area of inquiry that lends itself to multiple complementary approaches. By integrating neuroscientific, psychological, and artistic perspectives, each article of this Research Topic offers a unique insight into the interaction between creativity and human experience, enriching the discourse and suggesting new ways for research. This Research Topic serves not only as a meeting point for disciplines but also as a concrete demonstration of the transformative potential of an integrated approach, capable of profoundly influencing our understanding of the mind and its many expressions.

The first article explores the transformative potential of arts-based knowledge translation through the *Piece of Mind* project. By merging neuroscientific research with lived experiences, multimedia performances challenge societal misconceptions about neurodegenerative conditions such as Parkinson's and dementia. This approach exemplifies how combining scientific data with artistic media fosters empathy and understanding, producing emotionally engaging and accessible narratives. Audience feedback highlighted increased empathy and awareness, showcasing the effectiveness of interdisciplinary collaboration in combating stigma and enhancing public engagement. This project provides a model for bridging the gap between academic knowledge and public understanding, demonstrating the power of alternative dissemination methods (Kuhlmann et al.).

Castellotti et al. investigate the role of descriptive labels in digital and traditional art, focusing on their cognitive and emotional effects. Their findings indicate that descriptive materials enhance comprehension and evoke positive emotional responses, though their impact on aesthetic appreciation remains complex. Behavioral and psychophysiological data reveal that contextual information facilitates understanding and deepens the emotional experience of art. These insights highlight the interplay between cognitive and emotional responses in art perception, offering valuable guidance for curators, educators, and psychologists seeking to optimize art engagement.

An evolutionary perspective on aesthetic experiences is offered by Serrao et al., emphasizing the role of emotionally evocative art in fostering empathy and social cohesion. The authors argue that engaging with affective affordances in artworks has deep evolutionary roots, aiding individuals in navigating social and emotional contexts. By eliciting vicarious emotions, art strengthens emotional intelligence and facilitates social bonding. The study also addresses the paradox of deriving enjoyment from negative emotions in art, presenting a nuanced view of the adaptive value of such experiences. Art is positioned as a critical tool for emotional regulation and social learning, explaining its universal appeal across cultures and eras.

Gori et al. (2024) delve into the integration of artificial intelligence (AI) in artistic creation, examining its transformative potential for creative expression. AI serves as both collaborator and tool, challenging traditional notions of authorship and offering new perspectives on creativity. This study underscores how AI augments human creativity while opening avenues to explore cognitive and emotional processes underlying artistic generation. The interdisciplinary intersection of AI, psychology, and art raises ethical, philosophical, and practical questions that demand further investigation.

Li et al. explore the therapeutic applications of art and design in supporting individuals with visuospatial disorders. Their research highlights the benefits of tailored interface designs incorporating color and graphics, which enhance spatial orientation and cognitive rehabilitation. These findings illustrate the value of integrating artistic principles into therapeutic contexts to foster empowerment and emotional wellbeing. The study demonstrates how interdisciplinary collaboration can address complex health challenges through innovative visual environments.

Lastly, Tangredi et al. introduce artistic data visualization as a tool to enrich challenge-based learning environments. By integrating aesthetics with analytics, this approach enhances comprehension and engagement. Artistic visualizations make abstract data tangible, creating compelling narratives that bridge quantitative analysis with human-centered storytelling. The findings have significant implications for educators and policymakers, emphasizing the transformative role of art in fostering inclusive, engaging learning experiences.

This Research Topic demonstrates how art and psychology together provide a powerful framework for understanding and enhancing human experience. By integrating artistic creativity with psychological inquiry, it addresses the complexities of cognition, emotion, and social interaction, highlighting art's multifaceted role as a medium of expression, a therapeutic tool, and an educational resource.

Through an exploration of the neural and psychological mechanisms behind artistic engagement, these works reveal how art both shapes and is shaped by human cognition and emotion.

We hope readers find this Research Topic an inspiring resource, offering fresh perspectives on the evolving relationship between psychology and art.
